# Simulation Modeling and Metamodeling to Inform National and International HIV Policies for Children and Adolescents

**DOI:** 10.1097/QAI.0000000000001749

**Published:** 2018-07-11

**Authors:** Andrea Ciaranello, Annette H. Sohn, Intira Jeannie Collins, Claire Rothery, Elaine J. Abrams, Beth Woods, Pamela Pei, Martina Penazzato, Mary Mahy

**Affiliations:** * Department of Medicine, Medical Practice Evaluation Center, Massachusetts General Hospital, Boston, MA;; † Divisions of Infectious Diseases, Department of Medicine, Massachusetts General Hospital, Boston, MA;; ‡ TREAT Asia/amfAR—The Foundation for AIDS Research, Bangkok, Thailand;; § Medical Research Council Clinical Trials Unit at University College London (UCL), London, United Kingdom;; ‖ Centre for Health Economics, University of York, York, United Kingdom;; ¶ ICAP at Columbia University, Mailman School of Public Health, New York, NY;; # College of Physicians & Surgeons, Columbia University, New York, NY;; ** HIV and Hepatitis Department, World Health Organization, Geneva, Switzerland; and; †† Strategic Information and Evaluation Department, UNAIDS, Geneva, Switzerland.

**Keywords:** pediatric, adolescent, HIV, model, metamodel, guidelines

## Abstract

**Objective and Approach:**

Computer-based simulation models serve an important purpose in informing HIV care for children and adolescents. We review current model-based approaches to informing pediatric and adolescent HIV estimates and guidelines.

**Findings:**

Clinical disease simulation models and epidemiologic models are used to inform global and regional estimates of numbers of children and adolescents living with HIV and in need of antiretroviral therapy, to develop normative guidelines addressing strategies for diagnosis and treatment of HIV in children, and to forecast future need for pediatric and adolescent antiretroviral therapy formulations and commodities. To improve current model-generated estimates and policy recommendations, better country-level and regional-level data are needed about children living with HIV, as are improved data about survival and treatment outcomes for children with perinatal HIV infection as they age into adolescence and adulthood. In addition, novel metamodeling and value of information methods are being developed to improve the transparency of model methods and results, as well as to allow users to more easily tailor model-based analyses to their own settings.

**Conclusions:**

Substantial progress has been made in using models to estimate the size of the pediatric and adolescent HIV epidemic, to inform the development of guidelines for children and adolescents affected by HIV, and to support targeted implementation of policy recommendations to maximize impact. Ongoing work will address key limitations and further improve these model-based projections.

## INTRODUCTION

Computer-based simulation models serve several critical roles in informing HIV-related policies for children and adolescents. Clinical disease simulation models focus on important events that occur in individual patients; they capture details of HIV disease progression, care engagement and retention, and treatment outcomes, as well as their associated costs. Such models are often used to evaluate the clinical impact and cost-effectiveness of specific interventions, such as HIV testing or antiretroviral therapy (ART) strategies. Epidemiologic models focus on the impact of HIV on populations, including at the national and global levels. These models often capture clinical outcomes as well, and thus can be used to examine the impact of specific HIV-related interventions, but they additionally can provide estimates of numbers of new infections, people living with HIV, people in need of and receiving ART, and deaths due to HIV.

Both types of models offer insight into HIV policies in 5 key ways. First, they allow investigators to combine the best available data from multiple sources when no single source provides sufficient information: for example, clinical trial data to inform ART response, cohort data to inform disease progression risks, and epidemiologic and program data to inform numbers of children on ART. They similarly permit comparisons of multiple alternative strategies, even when no single study has compared all relevant options. Second, models can project beyond the time frame of clinical studies and estimate the impact of interventions well into the future. This is particularly important for children and adolescents, for whom the key outcomes of care offered now (eg, delay to ART switching after virologic failure) may not be seen for many years (eg, failure of later lines of ART due to accumulated drug resistance). Third, models require that all assumptions be made explicit; investigators can then evaluate the impact of each assumption and each uncertain data parameter. Through sensitivity analyses, investigators can determine which parameters most influence model outcomes, and in turn, policy recommendations based on these outcomes. Investigators can also identify the “threshold” values at which these policy recommendations would change and comment on the robustness of currently available data. When specific parameters are found to be influential but uncertain, further research can be prioritized toward improving data around these key parameters. Fourth, models can simulate important outcomes among populations not reached by HIV programs, for example, mortality among children not yet diagnosed with HIV or among children lost to follow-up. By characterizing outcomes for the complete population, models can highlight “treatment gaps,” offering a more comprehensive understanding of the pediatric HIV epidemic and the potential impact of improved service delivery. Finally, detailed simulation models can be used to develop metamodels, or simpler “models of the models,” which allow users to easily and quickly tailor model-based analyses to their own settings.

## CURRENT AND PREVIOUS USE OF MODELS TO INFORM PEDIATRIC HIV POLICY

Model-based analyses have informed pediatric HIV care in 3 key ways, each described below: projecting the potential impact of alternative guideline recommendations, estimating the magnitude of the pediatric HIV epidemic, and anticipating the need for pediatric ART and laboratory commodities. Here, we use “children” to refer to ages 0–14 years, “adolescents” for ages 10–19 years, and “youth” for ages 15–24 years.^1^

### Normative Guideline Development

Model-based analyses have been critical to inform normative guidance for children from the World Health Organization (WHO), in light of limited clinical trial and observational data for pediatric populations (Table 1). These analyses have examined the potential clinical and economic impact of new WHO guidelines if implemented at scale; they have also suggested ways to allocate limited resources among currently recommended practices to maximize health. For example, a model-based analysis demonstrated that lopinavir/ritonavir use in children younger than 3 years was both more effective and cost-saving over long time horizons compared with nevirapine, providing additional evidence to support the WHO 2013 recommendation for protease inhibitor–based ART as the preferred regimen for infants and young children.^2,3^

**Table 1.**
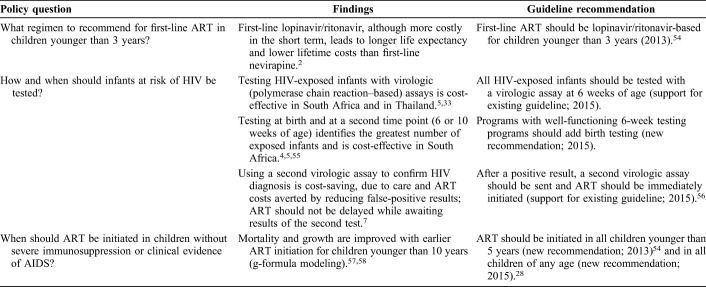
Selected Model-Based Analyses Informing WHO Guidelines for Children and Adolescents

Another domain that has benefited from model-based analyses is early infant diagnosis (EID). Multiple biologic, technological, and programmatic factors have been synthesized in a series of sophisticated mathematical models to inform the optimal approaches to diagnosing HIV infection in HIV-exposed infants. Lilian et al,^4^ using data from the South Africa's national EID program, concluded that the optimal algorithm to maximize the number of HIV-infected infants identified in South Africa included virologic testing at birth and 10 weeks of life. This work motivated the introduction of birth testing as part of routine care in South Africa. Francke et al^5^ used the Cost-effectiveness of Preventing AIDS Complications (CEPAC)-Pediatric model to simulate 4 EID strategies in South Africa; they demonstrated that testing at birth and at a second point in time (6 or 10 weeks of age) was cost-effective in South Africa. Both analyses underscored the importance of improving EID program coverage and quality to reduce mortality and morbidity among infants with HIV infection. These analyses went on to inform the WHO 2015 guidelines that recommended consideration of the addition of nucleic acid testing at birth to existing EID testing approaches.^6^ Additional examples are highlighted in Table 1.^7^

### National and Global Estimates for Children and Adolescents With HIV

Models are needed to estimate the national and global numbers of children and adolescents living with HIV.^1^ Although we would ideally count every child who is exposed to HIV and then test each child for infection, systems to collect those data are often not available; in addition, some women with HIV will not attend clinics or be captured in national health systems. Epidemiological models are therefore used to estimate the total, population-level numbers of women living with HIV and children infected with HIV. One example, Spectrum software, developed by Avenir Health and supported by UNAIDS, is the most widely used source of global estimates for the number of children living with HIV (CLHIV), new HIV infections, and AIDS-related deaths each year.^8^ These estimates are used by countries to inform policies, set national targets, and evaluate programmatic gaps, for example, to report on progress toward the UNAIDS Global Plan toward the elimination of new HIV infections among children.^9^ The estimates have also been used to advocate for new efforts to identify CLHIV who have not been diagnosed^10^ and to promote a multipronged approach to reducing new infections among children, including prioritizing ART coverage among breastfeeding mothers with HIV.^11^ Each year, the country estimates teams assemble updated local data, perform Spectrum simulations, and review Spectrum results with UNAIDS and other partners. Avenir Health investigators revise the Spectrum model structure based on rigorous recommendations from an international panel of experts in pediatric HIV, epidemiology, and modeling.^12^ With each annual revision, new estimates are generated for the entire epidemic, from 1970 through the present; estimates reported by UNAIDS therefore change annually for both current and previous years.^8^

### Forecasting of Pediatric HIV Medications and Commodities

Ensuring a continuous and reliable supply of pediatric antiretrovirals (ARVs) and diagnostic kits remains of paramount importance to ensure quality of care and access to HIV services for children. Forecasting the demand for these commodities is also critical to guide the development of new ARVs and diagnostic assays. Demand is currently forecast by extrapolating global estimates obtained from the Spectrum model, assuming a linear increase over time based on information about current ARV procurement orders.^13^ This approach has unfortunately failed to capture accelerated phases of pediatric scale-up or the nuances of different weight and age groups and different lines of treatment for children. New efforts are currently in place to revise these approaches and to fully address the unavoidable complexity of pediatric ARV treatment, particularly because new regimens and new formulations are being introduced for children and adolescents.^12,14^

## CHALLENGES AND OPPORTUNITIES IN EMPIRICAL DATA FOR MODELING PEDIATRIC HIV

The key types of data needed to improve model-based global pediatric HIV estimates are detailed below. We use the term “data” to reflect empirical data from clinical trials, observational studies, cohorts, and epidemiologic studies, which are used as inputs to models; we use the terms “results,” “estimates,” or “projections” to represent the output from these models.

### Need for Country-Specific Data and Recommendations

There is a clear need to adapt global pediatric HIV estimates and guidelines to local and regional levels. The most appropriate way to implement new recommendations, or to target interventions to specific populations, is likely to differ between settings that vary in terms of HIV prevalence, access to care, and health system infrastructure.^15^ Robust implementation research is often time-consuming, costly, or conducted in settings that may not be generalizable. For this reason, modeling—particularly cost-effectiveness analysis—is increasingly being used to explore the impact of different approaches to adapt global guidance for the greatest impact in a range of settings. However, many countries do not have the required data to use in existing models to accurately reflect their pediatric HIV epidemics. When local data are not available, investigators can use a combination of model-based analyses with best available data to approximate the local context. For example, not all countries collect the number of people on ART by age group; for UNAIDS estimates, countries therefore apply age-distribution data from research study sites or tertiary care facilities to the total number of children receiving ART nationwide.^16^ In Spectrum, a number of “default” values are provided when local data are missing; these include fertility reduction among HIV+ women, perinatal transmission probabilities, annual rate of CD4 decline without ART, survival among those on ART and those off ART, and the effectiveness of cotrimoxazole on reducing mortality.

### Need for Clinical Outcomes Among Perinatally HIV-Infected Children as They Age

Among the most important parameters that impact model-based global pediatric HIV estimates are survival, disease progression, and ART response among perinatally HIV-infected adolescents and perinatally HIV-infected youth (PHIVY) aged 10–24 years.^17,18^ Although empirical data exist for untreated children in the first few years of life, as well as for young children on ART, very little information is available about clinical outcomes for older children and youth in resource-limited settings.^10,19–22^ PHIVY have different outcomes than nonperinatally infected youth and adults, but detailed data suitable for model inputs are not yet available.^23,24^ Assumptions made in Spectrum and other models about survival with and without ART for perinatally infected children and PHIVY across the age spectrum could markedly influence country and global estimates of numbers of CLHIV and youth living with HIV, needing and receiving ART, and dying of HIV. For example, in the 2016 Spectrum revision, UNAIDS estimated that 1.8 million (range: 1.5–2.0 million) children aged 0–15 years had been living with HIV in the year 2015.^1^ In the 2017 revision, incorporating key improvements in input data and structure related to numbers of children perinatally infected with HIV and age at ART initiation (thus survival), Spectrum-based estimates indicated that there had been 2.2 million (1.8–2.7 million) CLHIV in 2015.^1^ Better data about clinical events for perinatally infected children will markedly improve these estimates and lead to greater consistency in yearly reports (Table 2). In addition, the proportion of all youth and adults with HIV who were perinatally infected is largely unknown. Spectrum revisions are underway to track PHIVY as they age into adulthood, and investigators in the International Epidemiology Databases to Evaluate AIDS (IeDEA) consortium and several other international research cohorts are collaborating to analyze clinical outcome data for PHIVY through age 24 to improve these Spectrum estimates.^1,15,22,23,25,26^

**TABLE 2.**
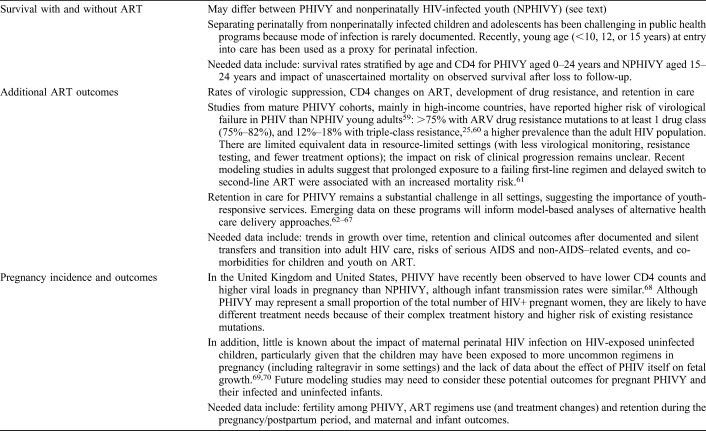
Key Data About Perinatally HIV-Infected Children and PHIVY Needed to Inform Epidemiologic Model Projections About Numbers of CLHIV and Youth Living With HIV, Receiving ART, and in Need of ART

### Need to Understand and Describe Data Quality and Biases in Available Data

The accuracy of modeled estimates of HIV prevalence and treatment outcomes depends on the reliability of the data used as model inputs. Accurate data may be more difficult to obtain for infants and children than for adults because of relatively smaller numbers and poorer coverage of testing and care. For example, because less than 50% of infants in need of EID are tested, many CLHIV are not diagnosed or initiated on ART until they are sick at older ages. The median age at entry into care among 0- to 19-year-olds in the IeDEA cohort was 6 interquartile range 2–12] years overall, and ranged from 4 years in West Africa (IQR: 2–9) and Asia-Pacific (IQR: 2–7) to 8 years in Latin America (IQR: 2–16).^1,27^ This delay in diagnosis means that many infants and children likely die before they can be diagnosed with HIV.^21,28^ Before the impact of this delay in diagnosis was included in Spectrum, model-generated estimates of pediatric HIV prevalence were likely too high, and projected HIV-related mortality rates were too low.^29^ Such corrections should be interpreted as necessary improvements in pediatric HIV-modeling methods because we seek to more accurately characterize this rapidly changing population. In addition, key questions remain about how to use available pediatric data, most of which describe children who started ART at older ages. These children likely experienced 2 competing types of “survivor bias”: they had more advanced disease at presentation to care than the entire cohort who “should” have been diagnosed and treated in infancy (perhaps, making them at greater risk of short-term mortality), but they also survived without ART for several years (suggesting slower disease progression that might reduce short-term mortality risk).^17^ These competing “survivor biases” may cause cohort-observed treatment responses and mortality risk to differ from the outcomes expected for all modeled children in Spectrum. Understanding the differences between “real life” programs, which provide model input data, and the cohorts that are simulated by models is essential to guide input selection and interpretation of model results.

## CHALLENGES AND OPPORTUNITIES IN MODEL-BASED METHODS IN PEDIATRIC HIV

Alongside efforts to improve the data used as model inputs, investigators are also working to improve the model-based methods themselves, building on innovative approaches in the fields of simulation modeling and cost-effectiveness analysis. These efforts are focused primarily on the need to compare model results with “real-world outcomes,” the need to improve transparency in model methods, and the need to improve access to user-friendly, manipulatable versions of complex models.

### Need for Comparison of Model Results With Real-World Outcomes and Cross-Model Comparisons

Despite intensive efforts to build models that reflect clinical and programmatic reality, we often lack a way to assess whether model-based predictions are “right.” Ideally, models would project anticipated outcomes, and after an appropriate period, we could compare these predictions with observed events. Although some model-predicted outcomes will not be fully observable (eg, mortality among all children not in care), other model-predicted outcomes may be suitable for such comparisons (eg, survival among children in care, numbers of children enrolled in programs, and HIV prevalence in sentinel testing projects). Model validation work has been facilitated by recent progress in electronic record systems to collect programmatic and epidemiologic data, improving data accessibility and quality. In addition, recently conducted population-based HIV impact assessments (PHIA surveys) have provided a unique opportunity to validate outputs of the Spectrum model.^30^ These comparisons have highlighted inconsistencies that have led to improvements in the structure and the data inputs of the Spectrum model.^29^

When suitable data are not observable (eg, undiagnosed HIV prevalence), comparing outputs from multiple models and investigating the reasons for any differences can provide critical feedback about the accuracy of both model structures and inputs. Ongoing work comparing Spectrum and the Thembisa epidemiologic model in South Africa is an example of this comparison for epidemiologic models, as is work by the HIV Modeling Consortium to estimate the impact of HIV treatment on HIV incidence using 12 different models for South Africa.^31,32^ Such cross-model comparisons are also useful when clinical disease simulation models examine similar clinical policy questions; although it is possible that multiple models are wrong, similar policy recommendations from multiple models may be reassuring for policymakers. Similar conclusions were reached, for example, by 2 models examining EID testing in Thailand and South Africa, and by 2 models investigating routine HIV testing for adults in the United States, with subsequent changes in WHO infant testing and US adult testing guidelines.^5,33–35^

### Need for Improved Transparency in Model Methods

Producers of both global health statistics and model-based policy recommendations have made important strides to allow for both data and model methods to be transparent. The GATHER statement is a set of transparency criteria for models used to generate global health estimates, and similar efforts have been encouraged for models used to support the normative guideline development.^36,37^ Many authors have called for modelers to make computer code freely available online, so that other investigators can replicate or build on published analyses. However, this may not be possible for highly complex models, for which users require intensive and ongoing training to understand interactions between components of the models, and learn to use updated features of the models to generate accurate results.^36,38–41^ For example, UNAIDS makes country Spectrum files, Spectrum software, and training manuals available online; for official users of the model, proficiency with these documents is supplemented by biennial, regional training workshops.^42,43^ The CEPAC model includes more than 30,000 lines of code and more than 28,000 input parameters; to avoid errors in inputs and execution, collaborating investigators undertake in-person trainings, usually 3–12 months in duration, at the team's research offices in Boston.^7,44,45^ Online tools and metamodeling methods can address these limitations in access.

### Need for Greater Access to Model Results and User-Friendly, Manipulatable Versions of Complex Models

Online- or spreadsheet-based calculators can permit policymakers and program planners to apply model-based analyses to their own settings. These tools allow users to input their own setting-specific values for key parameters (eg, HIV prevalence among women of childbearing age, prevention of mother-to-child transmission coverage, breastfeeding duration, or ART coverage among children). Because of the lengthy computing times for complex models, all simulations of interest must be conducted in advance to create such a calculator. Modelers need to anticipate the possible combinations of inputs that users might select, conduct simulations with all anticipated parameter sets, and generate a large, behind-the-scenes table of these model results. For example, an Excel-based tool allows country teams to understand the impact of prevention of mother-to-child transmission coverage on the mother-to-child transmission rate. This tool showed the importance of reaching all women with effective ARV regimens during the transition between the WHO 2006 guidelines and the “treat all” guidelines.^46^ A similar tool is under development to understand what interventions could allow a country to reach the target set for elimination of mother-to-child HIV transmission (defined as <50 new infant infections/100,000 live births).^47,48^ In addition, model developers can provide online access to tools that calculate key model inputs, for example, a calculator to derive age-based ART costs, using data from current price lists and HIV-specific adjustments to WHO and Centers for Disease Control and Prevention (CDC) weight-for-age standards.^47,49^

### “Metamodeling” Approaches to Permit User-Manipulable Tools

To expand beyond online tools that extract results from previously conducted model simulations, metamodeling methods allow for greater flexibility to rapidly tailor simulations from rigorous models to different setting scenarios. Developing a metamodel (also known as an emulator) involves running the complex simulation model for many combinations of model inputs and then using the information contained within these “runs” to create a simpler statistical model that can predict model outputs (eg, specific cost variables, life expectancy, and disability-adjusted life year for a given set of model inputs. There are many techniques that can be used to generate the statistical model that links inputs to outputs, including simple regression analysis or nonparametric regression using Gaussian processes.^50–53^ Once the statistical model has been developed, the model can then be evaluated very quickly for different input values. The parameter sets chosen by the user do not need to be the same as those used to develop the statistical model, allowing for much greater flexibility to examine setting-specific scenarios.

Metamodeling can support a number of important policy goals: (1) development of user-friendly tools for real-time use by decision makers, (2) rapid reanalyses of previous model-based work to inform application in additional contexts, eg, additional countries or subnational geographies, (3) quantification of the uncertainty around model results, and (4) further analysis to identify where evidence generation activities (eg, epidemiological surveys, intervention trials, pilot studies, or costing studies) would be of particular value to reduce the uncertainty around priority-setting decisions (further described below). Despite these potential advantages, there are relatively few applications of these techniques to models used to inform health care priority setting; although most of these are in the context of noncommunicable diseases and high-income settings, ongoing work by WHO and the CEPAC-Pediatric team is developing a metamodel to support decision-making for infant HIV testing.^50–53^

### Novel Methods to Understand the Impact of Uncertainty and to Prioritize Future Research

Most policy decisions are made under conditions of uncertainty, and model developers have an obligation to explain the magnitude and impact of this uncertainty to readers of their work. For example, Spectrum estimates are published with uncertainty bounds that highlight the impact of uncertainty in data inputs on the final projections of numbers of young people living with HIV. Quantifying the impact of uncertainty in model-generated results can serve 2 main purposes: (1) to assess our confidence in a chosen course of action by considering the degree to which variations in key model input parameters (eg, incidence of infections, treatment efficacy rates, or costs) affect the policy decision and (2) to assess the value of collecting additional information to better inform the decision by reducing the level of uncertainty in the current evidence base. Value of information (VOI) methods are a tool to quantify the consequences—both clinical and economic—that can result from uncertainty, as well as the likelihood of these consequences occurring. The potential value of further research is the expected improvements in health that can be gained by the associated reduction in the consequences of uncertainty (Table 3). VOI methods can be used, for example, to determine whether the information likely to be gained in a clinical trial is “worth” the cost of conducting the trial.^50–53^

**TABLE 3.**
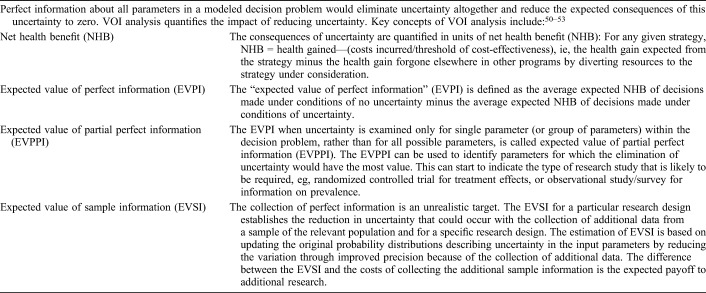
VOI Analysis—Technical Details

VOI typically requires a probabilistic sensitivity analysis (PSA). PSA involves specifying a distribution of values for all uncertain parameters; next, a large number of simulations are conducted, each time drawing a value for each parameter from the specified distributions. The variation in the model output over all these simulations reflects the impact of uncertainty in all these parameters simultaneously. PSA is often used, for example, to report the proportion of all simulations in which 1 strategy is favored over another. However, the processing time for repeated simulations of complex HIV models in HIV can preclude PSA, and thus VOI. Metamodeling can overcome these computational challenges; the metamodel itself can be used instead of the underlying simulation model to generate estimates of uncertainty in model results and VOI.^50–53^ The HIV Modeling Consortium is currently undertaking an investigation of alternative approaches to evaluating VOI in the context of HIV models and metamodels.

## CONCLUSIONS

Simulation models are critical to developing global and regional estimates of CLHIV, forecasting the need for pediatric ART and other commodities, and informing normative guidelines around pediatric HIV care when evidence gaps remain, as well as supporting targeted implementation of interventions to maximize impact. Both epidemiologic and clinical models incorporate the best available data from multiple sources to project outcomes that often cannot be observed in practice. All models make assumptions because data are limited. Modeling investigators must take seriously the obligation to make these assumptions transparent, test them through rigorous sensitivity and model validation analyses, and report the impact of this uncertainty on model results and resulting policy recommendations. Ongoing work to derive new data for CLHIV will continue to improve global pediatric HIV estimates. Novel approaches to make model-based analyses more accessible, through online tools and metamodels, will also improve the ability of model users to tailor model-based results for their own settings. Simulation models will become an additional tool to overcome evidence gaps and support accelerated action to reach global targets for CLHIV and adolescents living with HIV.
